# The transferome of metabolic genes explored: analysis of the horizontal transfer of enzyme encoding genes in unicellular eukaryotes

**DOI:** 10.1186/gb-2009-10-4-r36

**Published:** 2009-04-15

**Authors:** John W Whitaker, Glenn A McConkey, David R Westhead

**Affiliations:** 1 Institute of Molecular and Cellular Biology, University of Leeds, Leeds, West Yorkshire, LS2 9JT, UK

## Abstract

**Background:**

Metabolic networks are responsible for many essential cellular processes, and exhibit a high level of evolutionary conservation from bacteria to eukaryotes. If genes encoding metabolic enzymes are horizontally transferred and are advantageous, they are likely to become fixed. Horizontal gene transfer (HGT) has played a key role in prokaryotic evolution and its importance in eukaryotes is increasingly evident. High levels of endosymbiotic gene transfer (EGT) accompanied the establishment of plastids and mitochondria, and more recent events have allowed further acquisition of bacterial genes. Here, we present the first comprehensive multi-species analysis of E/HGT of genes encoding metabolic enzymes from bacteria to unicellular eukaryotes.

**Results:**

The phylogenetic trees of 2,257 metabolic enzymes were used to make E/HGT assertions in ten groups of unicellular eukaryotes, revealing the sources and metabolic processes of the transferred genes. Analyses revealed a preference for enzymes encoded by genes gained through horizontal and endosymbiotic transfers to be connected in the metabolic network. Enrichment in particular functional classes was particularly revealing: alongside plastid related processes and carbohydrate metabolism, this highlighted a number of pathways in eukaryotic parasites that are rich in enzymes encoded by transferred genes, and potentially key to pathogenicity. The plant parasites *Phytophthora *were discovered to have a potential pathway for lipopolysaccharide biosynthesis of E/HGT origin not seen before in eukaryotes outside the Plantae.

**Conclusions:**

The number of enzymes encoded by genes gained through E/HGT has been established, providing insight into functional gain during the evolution of unicellular eukaryotes. In eukaryotic parasites, genes encoding enzymes that have been gained through horizontal transfer may be attractive drug targets if they are part of processes not present in the host, or are significantly diverged from equivalent host enzymes.

## Abstract 2

Metabolic network analysis in multiple eukaryotes identifies how horizontal and endosymbiotic gene transfer of metabolic enzyme-encoding genes leads to functional gene gain during evolution.

## Background

Cellular metabolism is the network of chemical reactions that organisms use to convert input molecules into the molecules and energy they need to live and grow. Core metabolic processes and their enzyme catalysts are often conserved among the different kingdoms of life, which has allowed many species' metabolic networks to be automatically reconstructed from their genome sequences by the identification of homologs [1-5]. In addition to core metabolic processes, peripheral processes allow species to adapt to different environments - for example, metabolism of a rare sugar. This adaptation can be driven by the gain of genes encoding enzymes through horizontal gene transfer (HGT) [6], and this process has for some time been seen as an important aspect of prokaryotic evolution [7-9]. But as more eukaryotic genome sequences have become available, it has become clear that HGT has also occurred in the evolutionary histories of the eukaryotes [10].

HGT is likely to have had a more important influence upon the evolution of unicellular eukaryotes because there is no separate germline in which the transferred genes need to be fixed. Sources of HGT in eukaryotes include viruses, absorption from the environment, phagocytosis and endosymbiosis. HGT that accompanies endosymbiosis, termed endosymbiotic gene transfer (EGT), was important in establishing the eukaryotic organelles: the mitochondria and plastids. In addition to the primary endosymbiosis events that established plastids as eukaryotic organelles, multiple endosymbioses have occurred in unicellular eukaryotes [11,12]. An important example is the event, or events, that gave rise to the chromalveolates, in which a heterotrophic eukaryote gained a plastid through endocytosis of a plastid-containing red alga [13]. This brought together five genomes in one cell - two nuclear, two mitochondrial and one plastid - and with them came the opportunity for large scale EGT [14,15]. A further potential source of EGT in eukaryotes is from *Chlamydia *and may have occurred during the establishment of the primary plastid [16,17].

Among the unicellular eukaryotes are some important human and agricultural parasites, and consequently many have had their genomes sequenced, making comparative analysis of HGT possible within this group. Analysis of HGT in eukaryotic parasites offers interesting insights into their evolution. It is also of practical significance: horizontally transferred genes are often bacterial in origin, and thus more divergent from the host's eukaryotic equivalents than parasite genes of purely eukaryotic origin. They are therefore potentially good drug targets [18], owing to the increased likelihood of the discovery of parasite-specific inhibitors.

Methods of detecting HGTs from sequence data can be split into four categories: codon-based approaches that identify genes with a codon usage differing from the other genes in the genome [19,20]; BLAST-based approaches that identify sequences with high-scoring similarities to sequences from taxonomically distant species [21]; gene distribution-based approaches that compare the species that posses a gene to the accepted species phylogeny, allowing unusual patterns of gene possession that could be explained by HGT to be identified [6]; and phylogenetic approaches that construct phylogenetic trees and identify clades that differ from the expected organismal phylogeny [22,23]. Of the different methods of HGT detection, phylogenetic approaches offer the most power when studying HGT in eukaryotes. BLAST-based approaches have been shown to be misleading as the top BLAST hit is not always the closest evolutionary neighbor [24]; codon-based approaches are ineffective for ancient HGT events, such as EGTs, as over time sequences change to match the new genomic environment [25]; and gene distribution approaches rely strongly on good taxon sampling and the completeness of genome sequences.

Identification of all the HGTs in species' genomes allows the establishment and comparison of their transferomes (that is, all of the genes that the species has gained through HGT). Genes encoding metabolic enzymes are more likely to be involved in effective HGT from bacteria to eukaryotes than other classes of gene, because metabolic processes are more similar than, for instance, processes of genetic information processing [26,27]. There are several examples of the genes that encode metabolic enzymes being acquired through HGT in unicellular eukaryotes [14,28-31]. Metabolic enzymes can be positioned within well-defined biological processes and pathways, allowing the analysis of more detailed functional properties of the transferred genes that encode them, such as network connectivity. To investigate the extent of the horizontal transfer of genes that encode metabolic enzymes in unicellular eukaryotes, the metabolic evolution resource metaTIGER [32] was used. metaTIGER is particularly suited to this task because it contains 2,257 maximum-likelihood phylogenetic trees (with bootstrap analysis), each including sequences from up to 121 eukaryotes and 404 prokaryotes predicted to code for enzymes with specific Enzyme Commission (EC) numbers and located within reference metabolic networks. Furthermore, metaTIGER incorporates the program PHAT [22], a high-throughput tree searching program, which allows trees depicting HGT events to be easily identified. The high-quality trees and search tools provided by metaTIGER provide the foundation upon which this study is based.

## Results and discussion

### Levels of horizontal gene transfer in unicellular eukaryotes

To investigate the extent of HGT in unicellular eukaryotes, the metaTIGER phylogenetic tree database was searched for potential HGTs in the following groups of eukaryotes: *Plasmodium*,* Theileria*, *Toxoplasma*,* Cryptosporidium*,* Leishmania*, *Trypanosoma*,* Phytophthora*, diatoms,* Ostreococcus *and *Saccharomyces*. The species were considered in groups, each containing more than one species' genome sequence (groups are genera, with the exception of diatoms, which consist of two closely related genera, and *Toxoplasma*, which consists of two strains of the same species). Analysis was restricted to groups with more than one genome sequenced to prevent potential bacterial contamination in a single genome from influencing the results. *Saccharomyces *was included as a reference genus of non-parasitic, single-celled eukaryotic species believed to have never possessed a plastid-like organelle. Diatoms and *Ostreococcus *are photosynthetic and non-parasitic, while the remainder are important parasitic pathogens, including Apicomplexa (*Plasmodium*,* Theileria*,* Toxoplasma*, *Cryptosporidium*) and Trypanasomatids (*Leishmania*,* Trypanosoma*). The Apicomplexa, together with *Phytophthora *and the diatoms, lie within the eukaryotic supergroup of chromalveolates, believed to have gained a plastid by secondary endosymbiosis in the past, which is now lost in some cases. Detailed lists of the species used are included in Additional data file 1.

We refer to all putative gene transfers of plant, cyanobacterial and chlamydial origin as potential EGTs, while putative transfers of all other origins are referred to as HGTs. This is based on accepting the simplest explanation of events for gene acquisition; however, it should be made clear that phylogenetic trees only indicate a likely taxonomic source of genes and not the route through which they were acquired. Putative non-endosymbiotic transfers are split into two classes: 'recent HGTs', when the eukaryotic group being considered is the only genus of eukaryotes present in the clade upon which the prediction is based; and 'ancient HGTs', which occurred prior to the divergence of the genera concerned from eukaryotes in the same phylum - they are found when eukaryotes belonging to the same phylum are present in the clade upon which the prediction is based. Further details of gene transfer prediction can be found in Additional data file 1. Extensive EGT is known to have occurred between alpha-proteobacteria and the ancestor of the eukaryotes during the establishment of the mitochondria. Since this EGT is commonly believed to have occurred prior to the divergence of the eukaryotes being considered in this study [33], the transferred genes may be universal to them all and, therefore, difficult to identify as being of alpha-proteobacterial origin. For these reasons EGT of alpha-proteobacterial origin was not considered in this study.

**Figure 1:**
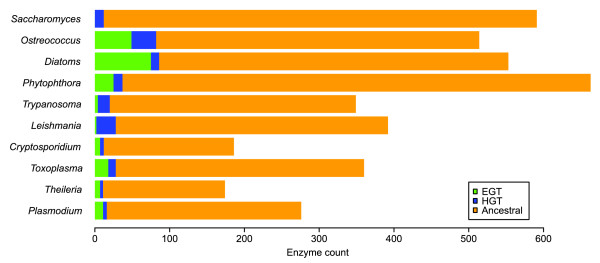
The predicted extent of the transfer of genes encoding metabolic enzymes. The bar chart shows the total number of enzymes that were identified as being present (high-confidence; see text) in each organism group. The numbers of enzymes whose genes were predicted as originating from EGT and HGT are indicated with green and blue, respectively.

When searching for trees depicting high-confidence HGT events, only clades with bootstrap support of 70% or above were considered (this has been shown to correspond to a high probability that the clade is correct [34]). We also retained lists of potential HGT events with less than 70% bootstrap support as a lower-confidence set. The trees resulting from the HGT searches were checked manually to ensure convincing evidence of E/HGT. The use of species groups containing more than one genome sequence, clades with bootstrap support of ≥ 70%, and the manual checking ensured that the high-confidence HGT assertions are as reliable as possible. Unless otherwise stated, results in this paper refer to the high-confidence E/HGT assertions. We consider these results to be an underestimate of the true level of EGT and HGT, since in some cases of E/HGT the sequences concerned will contain insufficient phylogenetic signal to assert this unambiguously [35]. Full details of the tree selection statements employed are contained in Additional data file 1. Figure 1 shows the overall levels of high-confidence E/HGT events in each species group, while a detailed listing of enzymes ordered according to Kyoto Encyclopedia of Genes and Genomes (KEGG) pathway is given in Additional data file 2.

As expected, no EGTs were found in *Saccharomyces*, while the number of predicted EGTs was greatest in two photosynthetic groups, *Ostreococcus *and the diatoms. The non-photosynthetic chromalveolates *Toxoplasma*, *Theileria *and *Plasmodium*, which have retained their plastids for non-photosynthetic metabolic processes, as well as *Cryptosporidium *and *Phytophthora*, which have lost their plastids, all have 4-5% of their enzymes originating from EGT and 2-3% of their enzymes originating from other HGTs. These transferred genes may represent viable drug targets, particularly if not found in the host genome. The trypanosomatids, *Trypanosoma *and *Leishmania*, are thought to have once possessed a plastid gained through secondary endosymbiosis [36]; however, only 1% of the enzymes found in their genomes were predicted as being EGTs potentially from this source. The high number of HGT genes encoding enzymes, 5-7% of all enzymes found, in thetrypanosomatids suggests that there are many potential drug targets of bacterial origin in these parasites (see below for further discussion). The EGTs that remain in species that have lost their plastid show that some EGTs have functions outside of the plastid, as observed in previous studies [14,15,37].

To our knowledge there have been no previous studies examining E/HGT in multiple species, with regard to the entire metabolic capacity, and performed on this scale. There have been studies of single species [14,28,30,38], and a study examining four apicomplexan species [39]. To assess our results, we compared them to previous work that looked at E/HGT in *Cryptosporidium parvum *[14]. This previous study found a total of 31 genes as potential HGTs, of which 20 were enzymes with specific EC numbers and can be compared to this work. Our results for *Cryptosporidium *comprise 12 high-confidence E/HGT predictions and another 21 lower-confidence predictions. Five of the high-confidence predictions made by this study were also made by the previous study. The predictions made by the previous study that were not high-confidence predictions in this study (n = 15): lacked the levels of bootstrap support needed to be considered high-confidence; did not appear to be HGTs based on evidence from our trees, which we attribute to the greater taxonomic coverage of available sequences in this later study; were very divergent genes (for example, genes in singleton OrthoMCL groups [40]) that were not assigned to specific EC numbers by the stringent criteria used in metaTIGER and, therefore, their sequences were not selected to be used in the metaTIGER phylogenetic trees; or, were not predicted as being present in both *Cryptosporidium *species. This comparison shows that assertions of HGT within eukaryotic genomes depend on confidence thresholds, and are subject to change as the taxonomic coverage of available sequences increases. It illustrates that our high-confidence predictions are likely to be underestimates, but supports their use in larger scale analyses in order to avoid the effects of potential false positive assertions.

### Horizontal gene transfers in the trypanosomatids that are potential drug targets

There is a great need for drug development against trypanosomatids. The large transferome identified in trypanosomes suggests a plethora of potential targets for drug development. This is exemplified by the enzyme pyruvate decarboxylase (4.1.1.1), whose gene is predicted to have been gained by horizontal transfer in *Leishmania*. Pyruvate decarboxylase has already been shown to be an effective drug target in *Leishmania tropica *as it serves as the target of the drug omeprazole [41,42]. Three new potential drug targets from the list of enzymes whose genes are predicted as having been horizontally acquired are: isopentenyl pyrophosphate isomerase (IPI; 5.3.3.2), isocitrate dehydrogenase (IDH; 1.1.1.42) and pyrroline-5-carboxylate reductase (PCR; 1.5.1.2).

IPI is used to convert isopentenyl diphosphate to dimethylallyl diphosphate in steroid biosynthesis, which is, in turn, used in the biosynthesis of farnesyl diphosphate. Blocking of a later step in the production of farnesyl diphosphate, through blocking farnesyl diphosphate synthase, has been shown to be effective in killing *T. cruzi in vitro *[43] and *in vivo *[44]. Humans have two copies of this IPI while *T. cruzi *has only one. The *T. cruzi *enzyme exhibits 28% identity with the 46 amino acids in the most highly conserved region of the enzyme when aligned with the human enzymes, suggesting that parasite-specific inhibitors could be developed.

Both humans and *L. major *have a mitochondrial and a cytoplasmic copy of the enzyme IDH. Mitochondrial IDH functions in the TCA cycle whereas the cytoplasmic enzyme is involved in regulating oxidative stress. The gene encoding the cytoplasmic copy of IDH was predicted as being a HGT in *Leishmania*. The enzyme is between 19% and 20% identical to the human ortholog when the most highly conserved region is aligned, suggesting that parasite-specific inhibitors could be developed. Cytoplasmic IDH is important in protection from oxidative stress in rats by supplying NADPH for the reduction of glutathione [45]. *Leishmania *do not use glutathione to protect themselves from oxidative stress but instead use other thiols, such as trypanothione [46,47], which also rely upon NADPH for their reduction. This suggests that targeting of *Leishmania*'s cytoplasmic IDH may increase its susceptibility to oxidative stress, which is one mechanism by which the host immune system combats these parasites.

PCR is the final enzyme in a pathway for the conversion of proline to glutamate, and is predicted to be the sole proline biosynthetic pathway in *T. cruzi*. There are two copies of the gene encoding *T. cruzi *PCR, which are 99% identical and are HGTs. Humans have six copies of this enzyme that are between 38% and 45% identical to the *T. cruzi *enzymes, suggesting that parasite-specific inhibitors could be developed.

### Double gene transfers

Three examples were observed where two genes encoding the same enzyme have been acquired from different sources within the same group of organisms: beta-ketoacyl-acyl-carrier-protein synthase I (2.3.1.41) in *Ostreococcus*, 2,4-dienoyl-CoA reductase (1.3.1.34) in the diatoms, and glucokinase (2.7.1.2) in *Phytophthora*. Beta-ketoacyl-acyl-carrier-protein synthase I in *Ostreococcus *was gained from cyanobacteria and *Chlamydia *and is involved in the plastid process of fatty acid biosynthesis, which explains its acquisition through EGT. 2,4-Dienoyl-CoA reductase in the diatoms was gained from both plants and gamma-proteobacteria and is needed if a *cis*-alpha-4 bond is present during beta oxidation, when Acyl-CoA molecules are broken down in mitochondria to generate Acetyl-CoA, which enters the Krebs cycle. Glucokinase in *Phytophthora *was gained from both plants and bacteroidales and is found in the KEGG pathways 'glycolysis/gluconeogenesis', 'galactose metabolism' and 'starch and sucrose metabolism'. It is possible that the glucokinases of different origins are optimal in different pathways. The gain and then retention of genes of multiple origins is an unusual observation within our results and there is no clear explanation for this. It is possible that the different copies could function in different pathways or locations within the cell; however, it could just be by chance that these multi-copy genes were gained from different origins, and then maintained, within these species.

### *Chlamydia *and endosymbiotic gene transfer

Recently, it has been suggested that a chlamydial endosymbiont facilitated the establishment of the primary plastid [16,17] in plants. To investigate this, the number of enzymes of chlamydial origin in *Ostreococcus *was examined (Table 1). Three enzymes of chlamydial origin were identified in *Ostreococcus*. In the diatoms, *Toxoplasma*, *Theileria *and *Plasmodium*, examples of EGTs from both plant and *Chlamydia *were found; these may represent enzymes whose genes were transferred from *Chlamydia *into plants and then transferred into the ancestor(s) of the chromalveolates. The EGTs of chlamydial origin support the idea that chlamydial endosymbiosis facilitated the establishment of the primary plastid. Two EGTs of chlamydial origin but not plant origin, which encode nitric-oxide synthase in *Phytophthora *and HMB-PP reductase in the four apicomplexans, were considered more likely to represent HGT than EGT. The gain of the HMB-PP reductase-encoding gene through horizontal transfer has been identified before [32] and seems to represent an orthologous replacement of an endosymbiotically transferred gene within the apicomplexan lineage.

**Table 1:** Relative predicted origins of EGTs

	*Plasmodium*	*Theileria*	*Toxoplasma*	*Cryptosporidium*	*Leishmania*	*Trypanosoma*	*Phytophthora*	Diatoms	*Ostreococcus*
Plant	4	4	11	5	1	3	20	41	NA
Cyano			1		1		2	1	45
Chlamy	1	1	1	1			1		3
Plant+cyano	3	1	2	1		1	2	28	NA
Plant+chlamy	3	1	3					4	NA
Chlamy+cyano									1

The number of EGTs of each putative origin is given for each species group; for example, for a gene's origin to be predicted as plant and cyanobacteria it must lie in a clade containing species of both these groups. The origins of the genes encoding the enzymes were predicted by using the metaTIGER tree searches (see text). Numbers refer to high-confidence predictions (clades with bootstrap values of 70 or above). Cyanobacteria and *Chlamydia *are abbreviated to cyano and chlamy, respectively. EGTs of cyanobacterial and plant, or chlamydial and plant origin represent bacterial EGTs into plants that have then been endosymbiotically transferred from plant into their new host - for example, during secondary endosymbiosis.

### Gene transfer and metabolic network connectivity

The idea that genes of related function might be co-transferred was investigated. To examine this, the number of connections (that is, metabolic network adjacency relationships corresponding to enzymes that catalyze consecutive steps in a pathway) between enzymes whose genes were acquired via horizontal transfer within the predicted metabolic network of each organism group was considered. This was done by calculating the average number of connections between enzymes whose genes had been acquired through horizontal transfer, and comparing this to the distribution of connection numbers between the same number of enzymes chosen at random from the group metabolic network. This randomization test was used to assess statistical significance (Additional data file 3). The degree of network connectivity between enzymes encoded by genes gained through EGT in the chromalveolates and *Ostreococcus *was found to be significantly greater than random, as would be expected since many chromalveolates and *Ostreococcus *still possess plastids containing complete plastid-specific pathways of endosymbiotically acquired genes. However, *Cryptosporidium *and *Phytophthora*, which have now lost their plastids, also show levels of connectivity between enzymes encoded by genes gained through EGT that are significantly greater than random. This shows that pathways, or at least pairs of connected enzymes that have functions outside the plastid, have been transferred during endosymbiosis.

The number of connections between enzymes encoded by genes acquired from bacteria was not found to be significantly greater than random in any species group. However, in *Leishmania *and *Ostreococcus*, where HGT is at the highest level, the network connectivity is approximately three times greater than the random value (with *P*-values of 0.065 and 0.054, respectively), suggesting a weak tendency towards the gain of genes whose protein products are connected within the metabolic network. It is possible that more statistically significant connectivity is masked to some extent by our requirement for high-confidence HGT assertions.

### Gene transfer and network complexity

Previous work on HGT between prokaryotes from a network perspective has determined that genes encoding proteins involved in complex systems are less likely to be transferred than those that are not [48]. In particular, this work found that 'informational genes' (those encoding proteins in transcription, translation, and related processes) were less likely to be transferred than 'operational genes' (for example, house-keeping genes). Since the analysis of E/HGT presented in this study focuses on metabolic enzymes, most of which are 'operational genes', it is not possible to investigate if this hypothesis holds true in eukaryotes. However, related work has considered HGT in the evolution of the *Escherichia coli *metabolic network, and found that genes that encode enzymes located at the periphery of the network are more likely to be gained through HGT than those in the center of the network [6]. To investigate if a similar trend was present in the E/HGTs predicted in this study, the average number of connections between an enzyme and other enzymes (within the metabolic network) was compared between E/HGTs and ancestral genes. Our analysis found no link between the number of connections and the origin of a gene encoding an enzyme (results not shown). The lack of observed difference might be due to the large number of parasites included in this study, which generally evolve through reductive evolution or gain-of-function for parasitism. Also, the E/HGT events being examined in this study are very ancient in comparison to the HGT events by which prokaryotes continually adapt their metabolic networks to their environment [49-51] and, therefore, have had more time to become more fully incorporated into the metabolic network.

### Enrichment analyses

Enrichment analysis was carried out to investigate if the genes encoding enzymes from particular functional categories are more likely to have been acquired through HGT. The functional categories considered were enzymes in the same KEGG map group (representing broad metabolic categories of KEGG maps), KEGG map (a smaller category of interconnected metabolic pathways) or KEGG module (representing defined pathways within KEGG maps); enzymes matching in EC number up to levels 1, 2 or 3; and enzymes using the same co-factors. For each functional category, the proportion of genes within each category resulting from E/HGT was compared with the proportion of E/HGTs over all categories and statistical significance was assigned using the hypergeometric distribution (although some of the functional groups contain very few enzymes, rendering statistical significance unlikely).

EGTs and HGTs were considered separately for each of the groups of species. The results of enrichment using the EC number levels and co-factors found very few significant results, suggesting that there is no underlying trend for enzymes with particular molecular functions to be transferred. The statistically significant results of the KEGG map group, KEGG map and KEGG module enrichment analysis are presented in Table 2. Additionally, the complete results of all five types of analysis are available in Additional data files 4 and 5.

**Table 2:** Biological pathways that are significantly enriched with E/HGTs

Type	Functional category	Species group	Total enzymes	EGT	HGT
Map group	Lipid met	*Ostreococcus*	64	10 (2.0*)	3
Map group	Lipid met	*Plasmodium*	36	6 (3.2^†^)	-
Map group	Lipid met	*Toxoplasma*	52	9 (3.7^†^)	-
Map	Bsyn of steroids	Diatoms	22	7 (2.7^†^)	-
Map	Bsyn of steroids	*Ostreococcus*	18	5 (3.5*)	-
Map	Bsyn of steroids	*Theileria*	7	2 (7.0*)	-
Map	Fatty acid bsyn	*Toxoplasma*	4	2 (10.7*)	-
Map	Fatty acid bsyn	*Plasmodium*	5	2 (7.8*)	-
Map	Fatty acid met	*Trypanosoma*	6	2 (10.1*)	-
Module	C5 isoprenoid bsyn, non-mevalonate	Diatoms	7	4 (4.3^†^)	-
Module	C5 isoprenoid bsyn, non-mevalonate	*Theileria*	6	2 (8.6*)	-
Module	C5 isoprenoid bsyn, non-mevalonate	*Plasmodium*	7	2 (6.4*)	-
Module	Fatty acid bysn, elongation	*Ostreococcus*	3	2 (6.6*)	-
Module	Fatty acid bysn, elongation	*Plasmodium*	3	2 (15.0^†^)	-
Module	Fatty acid bysn, elongation	*Toxoplasma*	3	2 (11.1^†^)	-
Map group	Carbohydrate met	*Cryptosporidium*	65	8 (2.6^†^)	2
Map group	Carbohydrate met	*Phytophthora*	195	22 (2.8^†^)	5
Map group	Carbohydrate met	*Plasmodium*	82	10 (2.4^†^)	2
Map group	Carbohydrate met	*Theileria*	61	6 (2.4*)	1
Map	Galactose met	*Phytophthora*	8	2 (6.3*)	1
Map	Glycolysis/Gluconeogenesis	*Phytophthora*	19	4 (5.3^†^)	1
Map	Glycolysis/Gluconeogenesis	*Plasmodium*	13	3 (4.5*)	-
Map	Pentose phosphate pathway	*Phytophthora*	12	3 (6.3^†^)	1
Map	Pyruvate met	*Cryptosporidium*	8	2 (5.3*)	-
Map	Pyruvate met	*Plasmodium*	13	3 (4.5*)	1
Map	Pentose and glucuronate interconversion	*Leishmania*	6	-	3 (8.2^†^)
Map	Starch and sucrose met	*Cryptosporidium*	11	2	2 (7.7*)
Map	Starch and sucrose met	*Phytophthora*	22	5 (5.7^†^)	1
Module	Glycolysis	*Phytophthora*	8	2 (7.0*)	-
Map group	Energy met	*Ostreococcus*	54	9 (2.1*)	2
Map	Carbon fixation	*Cryptosporidium*	8	2 (5.3*)	-
Map	Carbon fixation	*Ostreococcus*	19	5 (3.3*)	-
Map	Nitrogen met	*Toxoplasma*	11	-	2 (6.8*)
Map	Reductive carboxylate cycle	*Leishmania*	6	-	2 (5.5*)
Map group	Other AAs	*Leishmania*	32	-	5 (2.6*)
Map group	Other AAs	*Phytophthora*	54	1	4 (3.6*)
Map	Arginine and proline met	*Ostreococcus*	10	-	3 (5.3*)
Map	Glutamate met	*Plasmodium*	15	-	2 (6.6*)
Map	Glutathione met	*Leishmania*	10	-	3 (4.9*)
Map	Lysine bsyn	*Toxoplasma*	6	1	2 (12.5^†^)
Map	Nicotinate and nicotinamide met	*Leishmania*	4	-	2 (8.2*)
Module	Chorismate bsyn, phosphoenolpyruvate + erythrose-4P = > chorismate	Diatoms	6	3 (3.8*)	-
Module	Histidine bysn, PRPP = > histidine	*Ostreococcus*	5	-	2 (7.1*)
Module	Lysine bsyn, aspartate = > lysine	*Toxoplasma*	5	1	2 (16.6^†^)
Map group	Cofactor and vitamins	Diatoms	70	15 (1.8*)	2
Map group	Cofactor and vitamins	*Leishmania*	28	-	5 (2.9*)
Map group	Cofactor and vitamins	*Ostreococcus*	65	13 (2.5^†^)	4
Map	Biotin met	*Saccharomyces*	4	-	2 (29.7^†^)
Map	Porphyrin and chlorophyll met	Diatoms	20	12 (5.1^†^)	-
Map	Porphyrin and chlorophyll met	*Leishmania*	4	-	2 (8.2*)
Map	Porphyrin and chlorophyll met	*Ostreococcus*	21	12 (7.1^†^)	2
Map	Thiamine met	Diatoms	3	-	2 (22.4^†^)
Module	Biotin bsyn, pimeloyl-CoA = > biotin	*Saccharomyces*	3	-	2 (31.4^†^)
Module	Heme bsyn, glutamate = > protoheme/siroheme	Diatoms	10	6 (4.5^†^)	-
Module	Heme bsyn, glutamate = > protoheme/siroheme	*Leishmania*	3	-	2 (10.3*)
Module	Heme bsyn, glutamate = > protoheme/siroheme	*Ostreococcus*	10	5 (4.9^†^)	1
Map group	Glycan bsyn	*Phytophthora*	9	1	2 (10.9*)
Map	Lipopolysaccharide bsyn	*Phytophthora*	5	1	2 (19.6^†^)
Map group	Xenobiotics biodegradation and met	*Ostreococcus*	15	-	3 (3.5*)
Map	Aminoacyl-tRNA bsyn	Diatoms	19	6 (2.7*)	1

Significant over-representation of E/HGTs in biological pathways is shown on the following levels: Map group, KEGG map group; Map, KEGG map; Module, KEGG module. 'Total enzymes' is the number of enzymes in the species group within the defined category; 'EGT' and 'HGT' are counts of the number of transferred enzymes in the category followed in parentheses by the over-representation statistic for E/HGTs in that category (the proportion of E/HGTs for the category divided by the proportion of E/HGTs over all categories). Only statistically significant over-representation is shown and is indicated by asterisks (95% level) and dagger symbols (99% level). Significantly enriched pathways are not listed if the pathways contained only one E/HGT. The pathways are grouped by the KEGG map group they belong to in the following order: 'lipid metabolism', 'carbohydrate metabolism', 'energy metabolism', 'amino acid metabolism' and 'metabolism of other amino acids', 'metabolism of cofactors and vitamins', 'glycan biosynthesis and metabolism', 'xenobiotic biodegradation and metabolism' and 'amino-tRNA biosynthesis'. Abbreviations used in the pathway names: AA, amino acids; bsyn, biosynthesis; met, metabolism.

The KEGG map group 'lipid metabolism' (Table 2) is significantly enriched with EGTs in *Ostreococcus*, *Plasmodium *and *Toxoplasma*. Additionally, the diatoms and *Theileria *have near significant enrichment for 'lipid metabolism' with enrichment scores of 1.526 and 3.488, respectively. An enrichment of EGTs in 'lipid metabolism' is found in all the species groups that still possess a plastid. This enrichment of EGTs is a result of aspects of 'lipid metabolism', such as the non-mevalonate isoprenoid biosynthesis and type II fatty acid biosynthesis pathways, which occur within the plastid. Accordingly, some of these processes are also significantly enriched at the more detailed KEGG map and KEGG module levels. An interesting consequence of *Plasmodium *having retained many EGTs in 'lipid metabolism' is that its plastid (which has now lost all photosynthetic activity) must be retained for the parasite's survival [52-55].

The KEGG map group 'metabolism of cofactors and vitamins' is enriched with EGTs in the photosynthetic alga, the diatoms and *Ostreococcus*. The enrichment in this KEGG map group is mainly due to enrichment in the KEGG map 'porphyrin and chlorophyll metabolism'. Additionally, *Ostreococcus *was significantly enriched with enzymes in the KEGG map 'carbon fixation'. Again, genes originating from EGT enrich a section of plastid metabolism; however, this time they are involved in photosynthesis. The KEGG module 'heme biosynthesis, glutamate = > protoheme/siroheme' was found to be enriched with EGTs in the diatoms and *Ostreococcus*. This module contains a pathway that is common to eukaryotes and prokaryotes and is used to produce heme from L-glutamate. It has previously been shown that diatoms and plants have a common origin of this pathway, which mainly originates from EGT, but with some genes originating from mitochondrial EGT and others being ancestral [56]. Our high-confidence results agree with the previous analysis in all but one case where the endosymbiotic transfer of the gene encoding hydroxymethylbilane synthase (2.5.1.61) into the diatoms was omitted owing to insufficient bootstrap support (57%). These results show the successful identification of enrichment in pathways involved in photosynthesis, plastid-related lipid metabolism and heme biosynthesis with EGTs, indicating that despite the conservative nature of the high-confidence EGT predictions, well-supported underlying patterns of gene transfer can be identified.

The KEGG map group of 'carbohydrate metabolism' is enriched with EGTs in *Cryptosporidium*, *Phytophthora*, *Plasmodium *and *Theileria*. In particular, *Phytophthora *and *Plasmodium *are enriched with glycolytic enzymes; *Phytophthora *is enriched with enzymes involved in 'starch and sucrose metabolism', and *Cryptosporidium *and *Plasmodium *are enriched with enzymes involved in 'pyruvate metabolism'. Two important enzymes that feature in several KEGG maps, and in particular glycolysis, are pyruvate kinase and glucose-6-phosphate isomerase, and both their genes are predicted to have been acquired through endosymbiotic transfer in six organism groups. It is likely these were present prior to the secondary endosymbiosis event, suggesting these EGTs are examples of ortholog displacements. The enrichment of the KEGG map 'starch and sucrose metabolism' in *Phytophthora *is partly due to an enzyme involved in glucan metabolism and two enzymes involved in trehalose metabolism, which are discussed in detail below.

The enzyme 1-3-beta-glucan synthase (2.4.1.34), which produces 1-3-beta-glucan from UDP-glucose, was found to be endosymbiotically transferred into *Phytophthora*. Additionally, *Phytophthora *possess the enzyme 1-3-beta-glucosidase (3.2.1.58), which is responsible for breaking down 1-3-beta-glucan. *Phytophthora *use 1-3-beta-glucan for two essential functions: it is the most abundant polysaccharide in the *Phytophthora *cell wall, where it protects the cell from the plant's defense response and environmental stresses [57]; and it is also present in large amounts in the cytoplasm of *Phytophora*, where it is used as the principal storage polysaccharide used in sporulation, germination and infection [57].

Further functionally interesting endosymbiotic transfers into *Phytophthora *from within the KEGG map 'starch and sucrose metabolism' are two genes that encode the enzymes trehalose-6P synthetase (2.4.1.15) and trehalase (3.2.1.28). These are involved in trehalose metabolism; additionally, a third gene encoding a trehalose enzyme, trehalose-phosphatase (3.1.3.12), also appears to have been endosymbiotically acquired following manual inspection of its phylogenetic tree but was not in our high-confidence prediction list. Together these three enzymes form a reversible pathway that produces trehalose from UDP-glucose. Trehalose is a non-reducing disaccharide that is found in animals, fungi, plants and bacteria. It acts as a store of polysaccharide, but also provides resistance to a number of environmental stresses [36], including dehydration, extreme temperatures and damage by oxygen radicals. Stress resistance is highly relevant to *Phytophthora *during long periods of dormancy in soil, and while under attack by plant defense mechanisms, including damaging free radicals.

**Figure 2:**
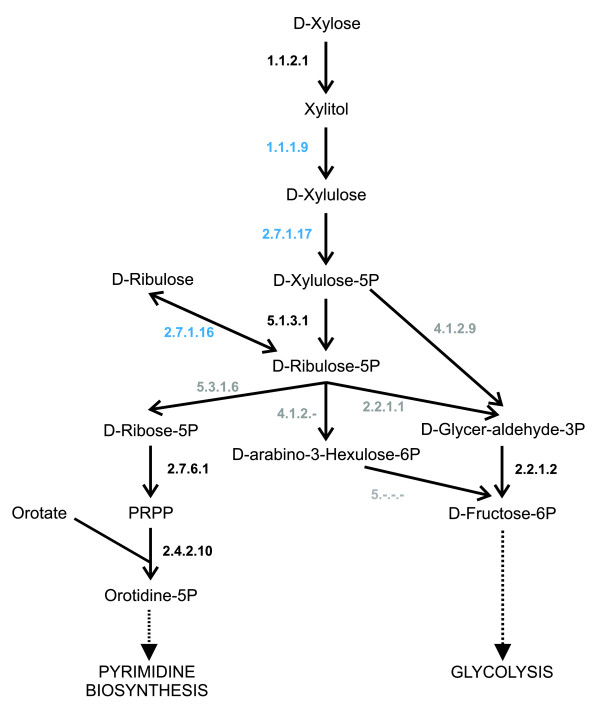
Xylose degradation in *Leishmania*. The figure shows a possible xylose degradation pathway in *Leishmania*. Enzymes shown in black are predicted as being present, the genes for enzymes shown in blue are predicted as being present and as being HGTs and the enzymes shown in grey are not predicted as being present. PRPP, 5-Phospho-alpha-D-ribose 1-diphosphate.

A recent review of *Leishmania *metabolism [58] suggested a bacterial origin of several enzymes that had been important to the parasite's metabolic adaptation. One of these enzymes is xylose kinase (2.7.1.17), which is part of the pathway 'pentose and glucuronate interconversion'. Our analysis predicted the gene encoding xylose kinase to have been horizontally transferred into *Leishmania*. Furthermore, another two genes, encoding enzymes from the same pathway, xylulose reductase (1.1.19) and ribulokinase (2.7.1.16), were also predicted as being gained through horizontal transfer, enriching the pathway 'pentose and glucuronate interconversion'. Inspection of the trees indicates that these enzymes originated from enterobacteria. With these enzymes and other less pathway-specific enzymes, a biochemical pathway can be reconstructed for *Leishmania *that produces ribulose-5P from xylose or ribulose (Figure 2). The ribulose-5P is used for *de novo *pyrimidine biosynthesis and glycolysis. Xylose may serve as a nutritional component for *Leishmania *during its vector stages as xylose is likely to be part of the diet of the sandfly.

The genes encoding three enzymes involved in heme biosynthesis, coproporphyrinogen-III oxidase (1.3.3.3) (high-confidence), protoporphyrinogen oxidase (1.3.3.4) (high-confidence) and ferrochelatase (4.99.1.1) (low-confidence), are suggested to have originated from HGT in *Leishmania*. This resulted in an enrichment of HGTs in the *Leishmania *'heme biosynthesis, glutamate = > protoheme/siroheme' KEGG module. Inspection of the trees containing the two high-confidence predictions suggests the enzymes were acquired from gamma-proteobacteria. The enzymes are likely to form a pathway allowing the biosynthesis of heme from porphyrin precursors; however, it is unclear at which life stage the pathway is operational [58].

The KEGG map 'glutamate metabolism' is enriched with three HGTs in *Leishmania*. One of these enzymes is glutathionylspermidine synthase (6.3.1.8), which produces mono-glutathionyl spermidine and is important in redox control in *Leishmania *[47]. A second enzyme, trypanothione synthase (6.3.1.9), is also important in redox control, and is encoded by a gene that is predicted to have been horizontally acquired in both *Leishmania *and the *Trypanosoma*. Trypanothione synthase is thought to have evolved from, and in some cases to have replaced, glutathionylspermidine synthase, which is now present as a pseudogene in *Leishmania major*, although it may still remain active in other trypanosomatids [59]. The resistance to oxidative stress that the products of these enzymes provide is very important to the pathogenicity of both the *Leishmania *and *Trypanosoma*. Manual inspection of the trees of glutathionylspermidine synthase and trypanothione synthase places the trypanosomatids in a clade that is separate and very divergent from the bacteria that comprise the rest of the tree. This suggests that rather than having been acquired via horizontal transfer, the genes encoding these enzymes may be ancestral genes that have only been retained in these basally diverging eukaryotes.

The pathway group 'glycan biosynthesis' in *Phytophthora *was enriched with HGTs as a result of two HGTs present within the KEGG pathway 'lipopolysaccharide (LPS) biosynthesis'. Additionally, a third enzyme in this pathway was identified as being encoded by a gene gained during endosymbiotic transfer. As LPS is an important virulence factor in pathogenic bacteria that has not previously been reported as being present in *Phytophthora *or any other eukaryotes outside the Plantae, further investigations of this pathway were carried out. Manual inspection of the phylogenetic trees of the two other enzymes that present in the metaTIGER 'LPS biosynthesis' pathway suggests that these enzymes might also have been acquired via gene transfers, although with low-confidence. As only 5 of the 30 enzymes in the KEGG 'LPS biosynthesis' pathway have EC numbers and enzyme models (that is, PRIAM profiles) and are therefore able to be detected by the SHARKhunt software, profiles for all 30 of the enzymes in the KEGG 'LPS biosynthesis' pathway were made (see Materials and methods for details). Searching the *Phytophthora *genomes with the 30 enzyme profiles identified 11 enzymes that are present in both genomes with E-values <10^-10 ^(see Additional data file 6 for full results).

**Figure 3:**
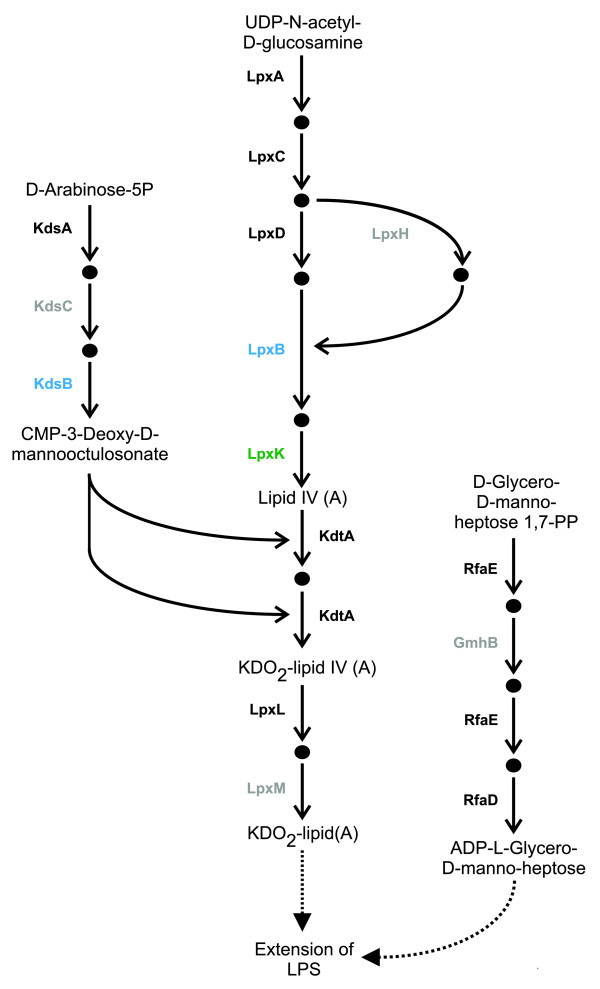
Lipopolysaccharide biosynthesis in *Phytophthora*. Enzymes that carry out reactions are labeled by *E. coli *gene name. The genes of the enzymes colored blue were predicted as being HGTs and the genes of the enzymes colored green were predicted as being EGTs. Enzymes colored black were predicted as being present in both *Phytophthora *genomes with profile E-values ≤ 10^-10^. Enzymes in grey were predicted as being present in at least one *Phytophthora *genome with E-values 10^-1 ^≥ E > 10^-10^.

Together these 11 enzymes carry out 13 of the 17 reactions (Figure 3) that are needed to form KDO_2_-lipid(A) and ADP-L-gylcero-D-manno-heptose. In Gram-negative bacteria these compounds form the minimal core structure of LPS [60,61]. The outer parts of LPS are more varied and hence the enzymes that catalyze their formation are likely to have diverged more than enzymes involved in the synthesis of the LPS core or may not be present in *Phytophthora*. In plant pathogenic Gram-negative bacteria, LPS is important for virulence as it reduces bacterial membrane permeability and sensitivity to antibiotics and antimicrobial peptides [62-64]. Additionally, it may play a role in attachment to plant surfaces [60,65,66]. Given that *Phytophora *is also a plant pathogen and will also have to cope with attacks from plant hosts, it is possible that its LPS may have a similar protective or attachment function.

## Conclusions

The metabolic evolution resource metaTIGER has successfully been used to construct a high-confidence dataset of enzymes whose genes are predicted to have been acquired through HGT in ten groups of unicellular eukaryotes. This collection of high-confidence predictions has allowed the transferomes of metabolic genes belonging to these organisms to be compared, providing new insight into their evolutionary histories. As expected, genes encoding enzymes involved in plastid metabolism were identified as EGTs, but more interestingly, other unexpected examples were identified. The unexpected examples of transfers included genes encoding enzymes that form previously unreported pathways in medically and agriculturally important pathogens. The gain of these pathways, via HGT, may have been an essential evolutionary step in their adaptation to a parasitic lifestyle. If the enzymes' functions are essential, then they could provide targets for future drug development. It is important to note, however, that genome sequencing in general has been biased towards pathogenic organisms, and the finding of E/HGT in pathogenicity-related pathways may reflect this.

During putative HGT prediction very stringent selection criteria were used. This means the results presented can be treated with confidence. However, it also means that the levels of HGT presented here are likely to be a conservative estimate of the actual levels of HGT that may have occurred. This is unavoidable as the sequences of many enzymes do not contain strong enough phylogenetic signal for reliable phylogenetic reconstruction. One possible cause of this, which has been recently highlighted, is horizontal transfer involving only parts of genes [67]. A greater understanding of species' transferomes would be gained if this work was expanded to incorporate genes of all functions. However, such work may encounter problems when the genes being considered are less functionally conserved than enzymes, making true ortholog identification much more difficult.

## Materials and methods

### Prediction of HGT enzymes

The transferred enzymes were predicted by using the metaTIGER web site [32]. metaTIGER is a metabolic evolution resource that contains the predicted metabolic capabilities of 121 eukaryotes. These were predicted with the program SHARKhunt [1], a high-throughput genome metabolic annotation program based on enzyme sequence profile searches. The enzyme profiles are based upon alignment of the amino acid sequences of conserved regions of genes of known function (EC number). These are used to search genomes using a combination of two sensitive bioinformatics techniques, PSI-BLAST and hidden Markov models, which means distant homologs can be detected in highly diverged organisms. Also incorporated into the metaTIGER site are 2,257 maximum-likelihood phylogenetic trees, which also include sequences from 404 prokaryotes. The trees only include sequence matches to enzyme profiles with E-values <10^-30^. If there is more than one sequence from a particular genome with an E-value <10^-30^, only the sequence with the lowest hit is included in the tree. These selection criteria aim to exclude paralogous genes as far as possible, and to ensure that trees are made only from orthologous sequences with specific EC numbers. The sequences were aligned using MUSCLE [68] and the trees were produced using the maximum-likelihood method PhyML [69]. Each of the trees was bootstrapped for 100 replicates allowing the confidence of putative E/HGT clades to be assessed. The phylogenetic analysis program PHAT [22] is incorporated into the site and was used in this study to identify the putative HGT events. Details of the PHAT selection statements that were used in HGT identification are given in Additional data file 1. All the predictions made were checked by inspection of the phylogenetic tree.

### Connectivity analysis

To investigate the degree of connectivity between the putative E/HGT, a predicted metabolic network for each of the species groups being considered is required. This was obtained from the KEGG reference metabolic network (constructed by parsing all the enzyme binary relations from the KEGG KGML files [3,70]) and retaining only those network connections involving enzymes predicted to be present in the species. This network was then used to find the average number of connections between the transferred enzymes. To assess statistical significance, the random distribution of enzyme connectivity was obtained from 10,000 random samples of the same number of enzymes from the network. To compare the number of connections (within the metabolic network) between enzymes gained through E/HGT and ancestral enzymes the average number of connections was calculated for each type within each species in the metabolic network.

### Enrichment analyses

To investigate if the genes encoding enzymes of particular biological or molecular functions are more prone to HGT, enrichment analyses were carried out within enzyme functional groups. These functional groups were based on the first three levels of the EC hierarchy, the use of particular co-factors and the division of the KEGG metabolic network into map groups, maps and modules. KEGG maps gather a number of interconnected and related metabolic pathways, map groups are sets of related maps, and KEGG modules are a set of defined pathways with each map. Within each functional group of enzymes, the proportion of HGTs was compared with the proportion of HGTs over all groups to identify enrichment (HGTs in the pathway/Total enzymes in pathway)/(Total HGTs in species group/Total enzymes in species group). Statistical significance was assessed using the hypergeometric distribution.

### Further investigation of lipopolysaccharide biosynthesis in *Phytophthora*

A number of enzymes in the KEGG 'LPS biosynthesis' pathway are not represented by sequence profiles in the SHARKhunt/PRIAM resources because they do not yet have EC numbers. For these cases, all protein sequences for each of the KEGG ortholog groups in the 'LPS biosynthesis' pathway were obtained from KEGG [3]. For each of the KEGG ortholog groups sequence profiles were made using SHARKmodel [1] and these were used to search the genomes of *P. sojae *and *P. ramorum*.

## Abbreviations

EC: Enzyme Commission; EGT: endosymbiotic gene transfer; HGT: horizontal gene transfer; IDH: isocitrate dehydrogenase; IPI: isopentenyl pyrophosphate isomerase; KEGG: Kyoto Encyclopedia of Genes and Genomes; LPS: lipopolysaccharide; PCR: pyrroline-5-carboxylate reductase.

## Authors' contributions

JWW conceptualized the study, carried out the research, analyzed the data and wrote the manuscript. DRW conceptualized the study, assisted with analysis and provided advice and revisions when writing the manuscript. GAM contributed expert knowledge of metabolism and parasitology and provided advice and revisions when writing the manuscript. All authors read and approved the final manuscript.

## Additional data files

The following additional data are available with the online version of this paper: a document including details of the horizontal gene transfer prediction (Additional data file 1); an Excel table of predicted enzymes and gene transfers ordered by pathway (Additional data file 2); an Excel table showing analysis of network connectivity and gene transfer (Additional data file 3); an Excel table on EGT enrichment analysis (Additional data file 4); an Excel table on HGT enrichment analysis (Additional data file 5); a document including results of searching for the KEGG LPS gene in *Phytophthora *(Additional data file 6).

## Supplementary Material

Additional data file 1 Details of the organism groups and PHAT selection statements used to identify the putative gene transfers. Click here for file

Additional data file 2 The table shows all of the enzymes that are predicted as being present in each of the organism groups. Enzymes that are not predicted as being gene transfers are shown in orange, EGT enzymes are shown in green, HGT enzymes are shown in blue and double transfers are shown in yellow. The enzymes are grouped by KEGG pathway. Click here for file

Additional data file 3 For each organism group and gene transfer type (EGT and HGT) the following information is given: the number of enzymes predicted as being gene transfers; the number of these enzymes present within the KEGG metabolic network; the average number of connections between the nodes within the KEGG metabolic network; the average number of connection between the same number of enzymes calculated over 10,000 random samples; a *P*-value and a Z score based on these random samples. Then, for each of the gene transfer types, *t*-tests and Wilcoxon signed-rank tests are given to calculate the probability that the transfers are more connected than random over all the species groups. Click here for file

Additional data file 4 The EGT enrichment analysis for map groups, KEGG maps, KEGG modules, EC number and co-factors is given. For map groups and the first EC number tier both over- and under-representation are shown and for all other enrichment analysis only over-representation is shown. For all enrichment types the following information is given: the number of EGT enzymes of that type for the given organism; the total number of enzymes of that type from the given organism; a *P*-value corresponding to the probability of the level of representation; and an enrichment score. The *P*-values were calculated using the hypergeometric distribution. For KEGG modules the number of additional low confidence EGT predictions is also shown. The low-confidence predictions lack the bootstrap support and manual inspection that the high-confidence predictions have. Click here for file

Additional data file 5 The same as Additional data file 4 except showing other HGTs. Click here for file

Additional data file 6 LPS biosynthesis enzymes that had hits to either *Phytophthora *genomes are listed. Next to the *E. coli *enzyme name is the KEGG ortholog group ID and the EC number of the group. The E-value of the hit in each of the genomes is listed. Click here for file
