# Supplementary material for: The transferome of metabolic genes explored: analysis of the horizontal transfer of enzyme encoding genes in unicellular eukaryotes
Source: Genome Biol. 2009 Apr 15;10(4):R36. doi: 10.1186/gb-2009-10-4-r36 (PMC2688927; doi:10.1186/gb-2009-10-4-r36)
Supplement: Additional data file 1 — Details of the organism groups and PHAT selection statements used to identify the putative gene transfers. [file gb-2009-10-4-r36-S1.doc]

# The groups of species considered

In this study 10 different groups of species were considered.

*Plasmodium*: *Plasmodium berghei, Plasmodium chabaudii, Plasmodium falciparum, Plasmodium gallinaceum, Plasmodium knowlesi, Plasmodium reichenowi, Plasmodium vivax* and *Plasmodium yoelii*.

*Theileria*: *Theileria annulata* and *Theileria* *parva.*

*Toxoplasma*: *Toxoplasma gondii* GT1 and *Toxoplasma gondii* ME49.

*Cryptosporidium*: *Cryptosporidium hominis* and *Cryptosporidium parvum*.

*Leishmania*: *Leishmania infantum*, *Leishmania major* and *Leishmania braziliensis*.

*Trypanosoma*: *Trypanosoma vivax*, *Trypanosoma cruzi*, *Trypanosoma congolense* and *Trypanosoma brucei.*

*Phytophthora*: *Phytophthora sojae* and *Phytophthora ramorum*.

Diatoms: *Phaeodactylum tricornutum* and *Thalassiosira pseudonana.*

*Ostreococcus*: *Ostreococcus tauri* and *Ostreococcus lucimarinus*.

*Saccharomyces*: *Saccharomyces* *paradoxus*, *Saccharomyces* *mikatae*, *Saccharomyces* *kudriavzevii*, *Saccharomyces* *kluyveri*, *Saccharomyces* *cerevisiae*, *Saccharomyces* *castellii* and *Saccharomyces* *bayanus*.

In eight out of ten groups the species present are all members of the same genus. In the *Toxoplasma* group the members are two different strains of the same species. In the Diatom group the members are different genera but are member of the same class.

# Putative gene transfer identification

The metaTIGER phylogenetic trees [32] were searched to find five different kinds of putative HGTs: EGT from plants, EGT from cyanobacteria, EGT from chlamydia, recent HGT and ancient HGT. Plant EGT analysis was not carried out for the green alga *Ostreococcus* as this would have identified all of the species ancestral genes, which are not of relevance to this study. Gene transfer identification was carried out using the program PHAT [22] which searches large databases of phylogenetic trees for examples with defined clade structure, and which is incorporated into the metaTIGER site. The selection statements that were used for *Plasmodium* are given below (directly analogous statements were used for other groups). These selection statements constitute a way of screening a large database of trees for trees of potential interest; subsequent manual checking of the identified trees is necessary and was carried out, and unconvincing examples were rejected.

All of the EGT select statements required more than one member of the groups being considered to be present. This was done to avoid contamination in a single genome from causing false positives. The EGT statements also required more than one of the donor organisms to be present to prevent contamination in one of their genomes from affecting the results. In the two bacterial EGT statements clades are rejected if they contain more than 9 other bacteria. In the plant EGT statement they are rejected if they contain more than 9 bacteria other than *Chlamydia* or *Cyanobacteria* (the more than 9 other bacteria is denoted by a ‘*’ next to the word Bacteria in these selection statements). In all EGT statements clades were rejected if they contained a total of more than one archaea or eukaryotes which are not believed to have ever posed an organelle of cyanobacterial origin. More undesired bacteria were tolerated within a clade than undesired eukaryotes as the bacteria undergo high levels of HGT there are far more of these organisms present in the metaTIGER phylogenetic trees than the other organism types.

## Chlamydia EGT

(Plasmodium{>1}) & (Chlamydiae{>1}) & ((Fungi | Fungi/Metazoa group | Pelobiontida | Malawimonadidae | Mycetozoa | Entamoebidae | Acanthamoebidae | Lobosea | Archaea){<2}) & !((*Bacteria){>9})

## Cyanobacteria EGT

(Plasmodium{>1}) & (Cyanobacteria{>1}) & ((Fungi | Fungi/Metazoa group | Pelobiontida | Malawimonadidae | Mycetozoa | Entamoebidae | Acanthamoebidae | Lobosea | Archaea){<2}) & !((*Bacteria){>9})

## Plant EGT

(Plasmodium{>1}) & ((Viridiplantae | Rhodophyta | Glaucocystophyceae){>1}) & ((Fungi | Fungi/Metazoa group | Pelobiontida | Malawimonadidae | Mycetozoa | Entamoebidae | Acanthamoebidae | Lobosea | Archaea){<2}) & ((Proteobacteria | Firmicutes | Actinobacteria | Spirochaetes | Deinococcus Thermus | Thermotogae | Bacteroidetes | Planctomycetes | Chlorobi | Aquificae | Chloroflexi | Fusobacteria){<10})

Both of the HGT statements identified clades with more than one member of the groups being considered. This was done to avoid contaminating in a single genome from causing false positives. Both of the HGT statements require more than bacteria to be present in the clade. This is done to avoid eukaryote to prokaryote gene transfers being identified. The ancient HGT statement excludes clades which contain eukaryotic organisms outside of the kingdom which is being considered. The recent HGT statements then excludes clades which contain gene transfers which contain any eukaryotes which are not as closely related to the group as its members are to each other.

## Ancient HGT

(Plasmodium{>1}) & ((Fungi | Fungi/Metazoa group | Pelobiontida | Malawimonadidae | Mycetozoa | Entamoebidae | Acanthamoebidae | Lobosea | Diplomonadida | Heterolobosea | Parabasalidea | Cercozoa | Euglenozoa | stramenopiles | Viridiplantae | Rhodophyta | Glaucocystophyceae){<2}) & (Bacteria{>9})

## Recent HGT

(Plasmodium{>1}) & !(*Eukaryota) & (Bacteria{>9})
