# Supplementary material for: The transferome of metabolic genes explored: analysis of the horizontal transfer of enzyme encoding genes in unicellular eukaryotes
Source: Genome Biol. 2009 Apr 15;10(4):R36. doi: 10.1186/gb-2009-10-4-r36 (PMC2688927; doi:10.1186/gb-2009-10-4-r36)
Supplement: Additional data file 6 — LPS biosynthesis enzymes that had hits to either Phytophthora genomes are listed. Next to the E. coli enzyme name is the KEGG ortholog group ID and the EC number of the group. The E-value of the hit in each of the genomes is listed. [file gb-2009-10-4-r36-S6.doc]

| E. coli enzyme name | E.C. number | KO id | *P. ramorum* | *P. sojae* |
| --- | --- | --- | --- | --- |
| LpxA | 2.3.1.129 | K00677 | 2.90E-81 | 9.30E-79 |
| LpxB | 2.4.1.182 | K00748 | 1.80E-79 | 1.70E-79 |
| LpxK | 2.7.1.130 | K00912 | 1.50E-43 | 4.10E-42 |
| KdsB | 2.7.7.38 | K00979 | 1.10E-83 | 1.30E-78 |
| KdsA | 2.5.1.55 | K01627 | 4.00E-103 | 3.00E-105 |
| LpxL | 2.3.1.- | K02517 | 2.10E-29 | 1.30E-22 |
| KdtA | 2.-.-.- | K02527 | 3.60E-86 | 5.80E-85 |
| LpxC | 3.5.1.- | K02535 | 6.30E-87 | 5.30E-83 |
| LpxD | 2.3.1.- | K02536 | 3.50E-74 | 2.50E-77 |
| LpxM | 2.3.1.- | K02560 | 8.30E-04 | na |
| RfaB | 2.4.1.- | K02840 | na | 8.10E-07 |
| RfaF | 2.4.-.- | K02843 | 0.011 | 0.001 |
| RfaG | 2.4.1.- | K02844 | 1.40E-09 | 5.80E-09 |
| RfaL | 6.-.-.- | K02847 | 0.006 | na |
| RfaP | 2.7.-.- | K02848 | na | 0.026 |
| RfaQ | 2.4.-.-. | K02849 | 0.005 | 0.028 |
| RfaY | 2.7.-.- | K02850 | 8.70E-05 | 5.30E-05 |
| LpxH | 3.6.1.- | K03269 | 1.40E-05 | 9.70E-07 |
| KdsC | 3.1.3.45 | K03270 | 0.006 | 0.034 |
| RfaE | 3.1.3.45 | K03272 | 1.20E-15 | 2.40E-18 |
| GmhB | 3.1.1.- | K03273 | 3.30E-12 | 1.20E-08 |
| RfaD | 5.1.3.20 | K03274 | 8.50E-15 | 4.90E-15 |
| RfaK | 2.4.1.56 | K03280 | 1.90E-07 | 2.00E-07 |
